# Distinctive Evidence Involved in the Role of Endocannabinoid Signalling in Parkinson’s Disease: A Perspective on Associated Therapeutic Interventions

**DOI:** 10.3390/ijms21176235

**Published:** 2020-08-28

**Authors:** Tapan Behl, Gagandeep Kaur, Simona Bungau, Rishabh Jhanji, Arun Kumar, Vineet Mehta, Gokhan Zengin, Roxana Brata, Syed Shams ul Hassan, Ovidiu Fratila

**Affiliations:** 1 Department of Pharmacology, Chitkara College of Pharmacy, Chitkara University, Punjab 140401, India; kgagandeep060@gmail.com (G.K.); arundhiman431@gmail.com (A.K.); 2 Department of Pharmacy, Faculty of Medicine and Pharmacy, University of Oradea, 410028 Oradea, Romania; 3 National Agri Food Biotechnology Institute, Mohali, Punjab 140306, India; rj.kuk01@gmail.com; 4 Department of Pharmacology, Government College of Pharmacy, Distt. Shimla, Himachal Pradesh, Rohru 171207, India; vineet.mehta20@gmail.com; 5 Department of Biology, Faculty of Science, Selcuk University Campus, 42130 Konya, Turkey; biyologzengin@gmail.com; 6 Department of Medical Disciplines, Faculty of Medicine and Pharmacy, University of Oradea, 410073 Oradea, Romania; roxana.gavrila@yahoo.com (R.B.); ovidiufr@yahoo.co.uk (O.F.); 7 Shanghai Key Laboratory for Molecular Engineering of Chiral Drugs, School of Pharmacy, Shanghai Jiao Tong University, Shanghai 200240, China; Shams1327@yahoo.com; 8 Department of Natural Product Chemistry, School of Pharmacy, Shanghai Jiao Tong University, Shanghai 200240, China

**Keywords:** endocannabinoid system, endocannabinoids, cannabinoid 1 receptor, cannabinoid 2 receptor, Parkinson’s disease, neuroprotection

## Abstract

Current pharmacotherapy of Parkinson’s disease (PD) is symptomatic and palliative, with levodopa/carbidopa therapy remaining the prime treatment, and nevertheless, being unable to modulate the progression of the neurodegeneration. No available treatment for PD can enhance the patient’s life-quality by regressing this diseased state. Various studies have encouraged the enrichment of treatment possibilities by discovering the association of the effects of the endocannabinoid system (ECS) in PD. These reviews delineate the reported evidence from the literature on the neuromodulatory role of the endocannabinoid system and expression of cannabinoid receptors in symptomatology, cause, and treatment of PD progression, wherein cannabinoid (CB) signalling experiences alterations of biphasic pattern during PD progression. Published papers to date were searched via MEDLINE, PubMed, etc., using specific key words in the topic of our manuscript. Endocannabinoids regulate the basal ganglia neuronal circuit pathways, synaptic plasticity, and motor functions via communication with dopaminergic, glutamatergic, and GABAergic signalling systems bidirectionally in PD. Further, gripping preclinical and clinical studies demonstrate the context regarding the cannabinoid compounds, which is supported by various evidence (neuroprotection, suppression of excitotoxicity, oxidative stress, glial activation, and additional benefits) provided by cannabinoid-like compounds (much research addresses the direct regulation of cannabinoids with dopamine transmission and other signalling pathways in PD). More data related to endocannabinoids efficacy, safety, and pharmacokinetic profiles need to be explored, providing better insights into their potential to ameliorate or even regress PD.

## 1. Introduction

Endocannabinoids, also known as endogenous cannabinoids (eCBs), are the endogenous lipid-based retrograde neurotransmitters that comprise of cannabinoid receptors and imitate the psychomotor effect of *Cannabis sativa* [1,2,3]. The endocannabinoid system (ECS) is widely known for its ability to regulate numerous physiological roles (modulation of immune system, cognition, appetite regulation, motor function, and pain). In recent times, a variety of studies have been investigating the function of cannabinoids (CBs) in several pathological conditions, including the neurological diseases [1,4,5,6].

The considerable rise in the scientific interest of promising therapeutic benefits of cannabinoids have displayed a remarkable improvement in parkinsonian symptoms like dyskinesia or tremors [7,8]. Moreover, it has led to an increasing interest in research exploring the potential of these CBs and ECS as possible medical interventions for the treatment of various diseases, including neurodegenerative disorders [8]. The involvement of the cannabinoid 1 (CB1) receptor in ECS is a well expressed phenomenon in basal ganglia, which is reported to be affected in case of different motor dysfunctions and other neurodegenerative disorders including PD [9]. Additionally, the role of ECS in basal ganglia functioning and cortico-striatal pathway regulation has been explored competently in PD models [10,11,12,13]. PD is known as a progressive, chronic neurodegenerative disorder, which has become a considerable social and medical concern, while becoming a financial burden on public health systems in many countries.

The dopaminergic neuronal loss and α-synuclein aggregation in the Lewy bodies that invades the substantia nigra pars compacta (SNpc) are the major pathognomonic hallmarks of this disease [14,15]. Considered to be a multiplex of various environmental, genetic, and age-related factors, the aetiology of PD is still a widely disputed topic [9,16]. Although well-marked environmental or genetic factors tend to be correlated by several cases, the combination of unknown and unspecified genetic and environmental processes accounted for the majority of cases [17,18]. The deterioration of motor functions is easy cognoscible because of the following typical clinical observable symptoms in PD patients: rigidity, akinesia, bradykinesia, hypokinesia, stooped posture, postural instability, rest tremors, etc., all of them often existing. Cognitive impairment, olfactory dysfunction, psychiatric symptoms, and autonomic dysfunction are common non-motor features. Such motor functions are used to track the therapy response and assess improvement in PD patients [19,20,21]. However, levodopa remains the main symptomatic therapy of PD. Its persistent use in the initial years of the therapy is associated with motor fluctuations in 62% of patients and levodopa-induced dyskinesia (LID) that affects 91% of the patients, as reported by a 10-year prospective study in PD patients [22,23].

Thus, the ongoing study aims at employing newer non-dopaminergic substances that can prevent LID and alleviate motor symptoms. One such engrossing group of agents is represented by the CBs that have not only revealed their neuroprotective capability but also their potential to relieve motor and non-motor symptoms as detected in patients with PD, in various preclinical and clinical studies [23,24,25,26]. However, there is a shortfall of clinical data on the utilization of CBs in patients with PD but preclinical findings indicate that the modulation of the CB signalling pathway could incapacitate and improve motor impairments and symptoms [27,28,29]. The present review aims to lay out an overview of endocannabinoid and its probable influence for the treatment of parkinsonian symptoms, as well as for the expression of cannabinoid receptors in the symptomatology, cause, and treatment of PD progression (wherein cannabinoid signalling experiences alterations in the biphasic pattern and biochemical interactions between CBs, dopamine (DA), and ECS-targeted therapeutic interventions). Published literature data in the field were searched via the most well-known medical data bases (MEDLINE, PubMed, etc.), using specific key words in the topic of our manuscript, and resulting in 202 references mentioned at the final of this review type article.

## 2. The Endocannabinoid System

Notable advancement in our comprehension of the ECS had been established from the last 15 years. The ECS is a biological lipid-transmitting cascade that includes eCBs, molecules derived from arachidonic acid, and membrane phospholipids. The activity of CBs is expressed via Gi/o protein-coupled receptors, the CB1 receptor, and the cannabinoid 2 (CB2) receptor, being mediated via binding of the ligands to the metabotropic receptor; as well, other receptors are mentioned below, along with the enzymes and agents responsible for the synthesis, degradation, and transportation of eCB, which are essential elements of the body in both physiological and pathological aspects [30,31,32]. The detailed understanding of cannabinoid receptors, their specific locations and actions in the brain was followed by isolation of 2-endogenous arachidonic acid-derived ligands, anandamide/arachidonoyl ethanol amide (AEA), and 2-arachidonoyl glycerol (2-AG), the best-identified eCBs where the former was first to be isolated from porcine brain, and the latter obtained from canine intestines [32,33,34,35,36,37,38,39].

### 2.1. Cannabinoid Receptors (CB1 and CB2 Receptors) and Other ECS Associated Receptors

Cannabinoid 1 receptors (CB1 receptors) are crucial signalling mediators predominantly found in the peripheral and central neurons and are best characterized on both the gamma-aminobutyric acid (GABA) and glutamatergic neurons, possessing an excitatory or inhibitory activity. They are enabled in response to the depolarization regulating functions such as pain perception, memory, etc. [40,41,42,43]. Except for conventional neurotransmitters, eCBs are not retained in the synaptic vesicles; rather they act on presynaptic receptors in a retrograde manner after their synthesis and release on demand. It is assumed to be an essential aspect of the neuronal CB1 receptor element that modulates the neurotransmitter release, further establishing homeostasis by avoiding an extreme neuronal activity developing in the central nervous system (CNS) [40,41,44]. Higher levels of CB1 receptor are observed in the globus pallidus (GP), the molecular layer of the cerebellum, substantia nigra (SN), hippocampus, and caudate putamen [40]. The GP and SN (two protruding regions engaged in movement control) not only have the strongest CB1 receptor concentrations but rather the strongest eCB concentrations, specifically the N-arachidonoyl-ethanol amine (AEA) [10,45,46]. The CNS and supraspinal areas of the brain are extensively expressed with one of the primary receptors of CB compounds, i.e., the CB1 receptor involved in nociceptive transmission in the brain. The CB1 receptor is also found to be expressed in the frontal-limbic regions of the brain, which significantly regulate the emotional manifestations of the human brain [47]. CB1 receptor regulates a descending inhibitory pathway from the supraspinal level to the spinal cord nociceptive system via inhibiting GABA release [48,49]. At the spinal cord level, the CB1 receptor significantly mediates the noxious stimulation to the brain [50]. Furthermore, the CB1 receptor is also responsible for confining pain signal propagation, contributing to the peripheral analgesic effects [51].

The expression of CB2 receptors is primarily observed in the immune system such as macrophages, lymphoid organs, T and B cells, and immunocompetent cells at the peripheral regions and modifies numerous aspects such as lymphocytes proliferation, development of cytokines, and cell-mediated immune reactions. Indeed, scientific findings have shown that they often exist in CNS but at rates lower than the CB1 receptor. CB2 receptors situated on astrocytes and microglia, do not seem to play a major role in the cortico-striato-pallidal-circuit modulation. Instead, these are primarily upregulated in response to cytotoxic and neuroinflammatory injury [52]. However, the CB1 receptor is abundantly expressed in the basal ganglia and is actually denser in the substantia nigra (SN), indicating a probable role in the motor control movement [53,54]. Though, these nigrostriatal neurons do not express CB1 receptor themselves, they do express transient receptor-potential type-1 vanilloid (TRPV1) receptors that are activated by AEA [55].

Cannabinoid 2 receptor (CB2 receptor) activation mainly regulates microglial stimulation and recruitment and therefore plays a significant part in neuroinflammation, along with the inflammatory reaction identified in neurological disorders as demonstrated in animal models [14,40,56,57,58]. It could be specifically critical in controlling astrocyte action caused by various cytotoxic insults that could solely be caused by CB2 receptor stimulation or may include the CB1 receptor, individually or collectively with the CB2 receptor. Regardless of the type of CB receptor-associated, the advantages that are delivered seem to be correlated mostly with the trophic function performed by the glial cells (enhancements within the distribution of metabolic substrates to neurons). They may indeed promote the levels of anti-inflammatory mediators (transforming growth factor-β as pro-survival; TGF-β), interleukin (IL)-1 receptor antagonists, IL-10, or neurotropin production that could retrieve impaired neurons [59]. The CB2 receptor activation by microglial cells diminishes the generation of neurotoxic elements (i.e., tumour necrosis factor-α (TNF-α)), which is a key player in the pathology involved in brain injury. Activation of CB2 receptors restricts TNF-α development by suppressing nuclear factor-kappa B protein (NF-κB), a transcription factor that plays a vital role in monitoring the inflammatory reaction [59,60]. The latest evidence revealed the involvement of the CB2 receptor in some neural subpopulations in the brain, endorsing the potential role of the receptor in synaptic processing, though it has still not been established comprehensively. There is no confirmation about the stimulation of such neuronal CB2 receptor showing the neuroprotective effect, but they can appear as a reliable biomarker for neuronal dysfunction in neurodegenerative conditions such as PD [61,62,63,64,65].

Another receptor implicated in the movement control is a protein named transient receptor potential cation channel subfamily V member 1 (capsaicin receptor and vanilloid receptor 1, encoded TRPV1 gene), found in the dopaminergic neurons mainly basal ganglia circuit and sensory neurons. Endo-vanilloid-nociceptive stimuli particularly activate the molecular integrators, namely the TRPV1 receptors. TRPV1 communicates with the eCB, wherein AEA is one of the principal endogenous activators. Studies have also shown that the stimulation of vanilloid receptors can inhibit motor activity, indicating that TRPV1 receptors may perform a role in regulating motor function [66,67,68,69].

G protein-coupled receptor 55 (GPR55), an orphan G-protein receptor expressed widely in the brain (mainly in the striatum), is also a promising target in the PD therapy as it is thought to be involved in the motor function [70,71]. Even though, it lacks a classic CB binding pocket, GPR55 was regarded as “the 3rd CB receptor” and its signalling can be affected and activated by CBs [72,73,74]. In the PD mouse model, downregulation of the GPR55 expression was reported in the striatum and treatment with the abnormal-cannabidiol (Ab-CBD, a synthetic cannabidiol (CBD) isomer and GPR55 agonist) has shown a neuroprotective effect on dopaminergic neuronal cells and improved the motor behaviour [71,75]. An anatomical, molecular, and behavioural analysis of GPR55−/− mice models showed normal brain structure development and did not influenced ECS and motor learning. The mice displayed flaws in motor coordination, suggesting the involvement of GPR55 signalling in neurodegeneration and modulation of cytokines [76]. The symptomatic impact of Abn-CBD and two other GPR55 agonists (CID1792197 and CID2440433) was studied using the catalepsy test, which indicated GPR55 as a probable symptomatic target for PD [71].

### 2.2. Dopamine and Cannabinoid Interactions

**Figure 1:**
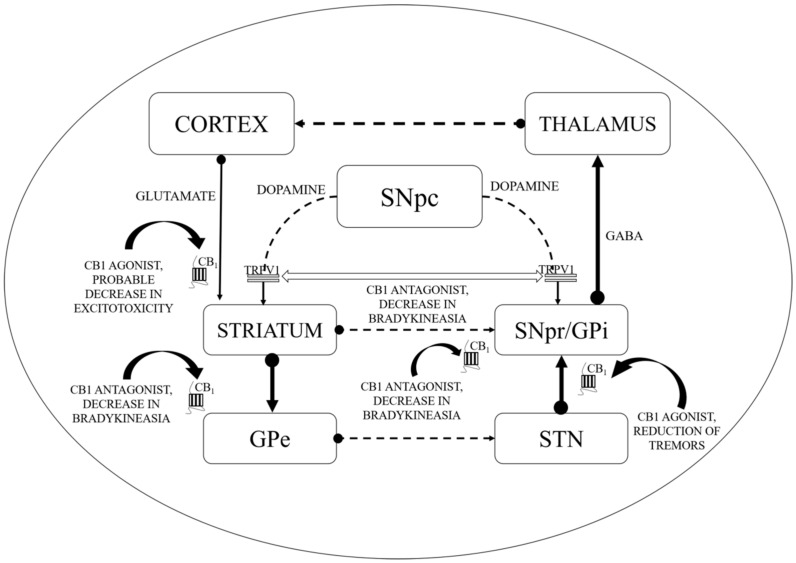
Basal ganglia circuitry and mechanisms depicting the cannabinoid (CB) targets in motor disability improvement in Parkinson’s disease (PD). CB1—Cannabinoid 1 receptor; GABA—γ-aminobutyric acid; GPe—Globus pallidus (external); GPi—Globus pallidus (internal); SNpc—Substantia nigra pars compacta; SNpr—Substantia nigra pars reticulata; STN—subthalamic nucleus; TRPV1—Transient receptor potential type-1 vanniloid; dotted line arrows—underactive; full line arrows—overactive.

Throughout the areas of the brain mentioned above that are significant for many of these neuropsychiatric disorders, CB-1R stimulation could render any of the following:

- Directly interacts and regulates the expression of dopaminergic neurons (by forming heteromers with dopaminergic receptors, as shown in Figure 1).
- Impedes the transduction of dopamine (DA) signals in CB-co-localized postsynaptic DA receptors.

CB1 receptors are not located on dopaminergic cells but influence the output of DA by modulating the release of neurotransmitters from projecting excitatory and inhibitory terminals via CB1 receptor stimulation [77,78]. As revealed by additional evidence, the existence of the CB2 receptor in nigrostriatal dopaminergic cells may permit the direct dopaminergic activity control via the endogenous CB mechanism (i.e., the random and elicited discharge, production, secretion of DA, etc.) [79,80]. ECS stimulation has been correlated with motor suppression and declined dopaminergic function (shown in Figure 1).

The heteromer of the CB1–dopamine 2 receptor (D2) creates a better example of G-protein switching within the heteromer. Thus, CB1 and D2 receptors are generally coupled with Gi/o proteins. Co-stimulation of CB1–D2 receptors in these cellular models leads to a protein-dependent activation of adenylyl-cyclase by Gs [81,82]. Several other researches described simple co-expression of D2 and CB1 receptors is likely to elicit the stimulation of adenylyl cyclase in response to CB1 receptor activation [83]. In various elements of striatal spine modules, D2 and CB1 receptor are colocalized. As stated above, D2 receptors are widely expressed in GABAergic dendritic spines. In addition, D2 receptors are also identified to be widely distributed in dopaminergic, GABAergic, and glutamatergic terminals in the striatal spine modules, wherein their stimulation suppresses the release of neurotransmitters [84,85,86]. Hence the probable localization of CB1–D2 receptor heteromeric may occur both pre- and post-synaptic (i.e., GABAergic enkephalinergic neuron dendritic spines) [87]. Experimental studies in rat striatal membrane provided extra strong evidence, demonstrating an intramembrane receptor–receptor interaction. CB1 receptor stimulation in these preparations declined D2 receptor affinity to DA [88,89,90]. These two features of the CB1–D2 receptor heteromeric, the antagonistic intramembrane interaction and G protein switching, would assume that activation of the CB1 receptor in functional experimental studies must antagonize the effects of striatal neurotransmission mediated by the D2 receptor. It is well established that agonists and antagonists of the CB1 receptor prevent and potentiate motor-mediated effects of D2 receptors, respectively [91,92,93]. Thus, it was proposed that the agonist-mediated motor-depressant effects of the CB1 receptor rely on their ability to prevent the neurotransmission of DA. Since motor activation induced by D2 receptor agonists is mostly dependent on postsynaptic D2 receptors, the behavioural studies strongly suggest that striatal CB1–D2 heteromers are localized in the dendritic spines of GABAergic enkephalinergic neurons. As indicated by this study, another locus where CB1–D2 heteromerisation could partially explain the CB1–D2 receptor interactions at the behavioural level are the terminals of the GABAergic enkephalinergic neurons in the GP [92,94].

Conventionally, limited eCB tone in hyperkinetic situations accompanies greater dopaminergic activity and the reversible pattern is noticed in hypokinetic motion disorders [95]. CBs could enhance the hypokinetic influence of DA-depleting agents in experimental PD models and lessen the incidence of drugs that produce DA receptor hyperstimulation [95,96]. Interaction between CB and DA appears to be much more complicated at the cellular level. In the striatum, the levels of eCBs are affected by the dopaminergic transmission initially as depicted by the higher levels of AEA following the stimulation of the D2 receptors as shown in Figure 1 [97]. Such phenomenon is based on either the suppression of its inhibition or the stimulation of its synthesis as proposed by the ability of the D2 receptor agonists to further modify the activity of the fatty acid amide hydrolase (FAAH) and *N*-acyl-phosphatidylethanolamine (NAPE) phospholipase D. Such activity of CBs stimulated by DA counteracts the action of D2 receptor in the striatum, indicating an inhibitory process focused at restricting the hyperkinetic effect of DA. To introduce extra entanglements, the observations that AEA generated by DA stimulation may intensify the effectiveness of D2 receptor activation, are also indicating cooperative behaviour of CB1 and D2 receptors [10,98,99,100]. However, suppression of GABA transmission through D2 receptors may slightly be avoided by the CB receptor blockade, indicating eCBs to serve as downward effectors for D2 receptors. While both CB1 and D2 receptors are presented on striatum at the GABA terminals, the intricate relationship between DA and eCBs clearly defines the reconfiguration of such systems in both experimental and idiopathic PD [97]. Earlier studies showed improved eCB behaviour in the basal ganglia of experimental PD along with increased levels of CB1 receptors, AEA, CB1 messenger ribonucleic acid (mRNA), and reduced CB clearance. Similarly, the cerebrospinal fluid of untreated PD patients was found to possess elevated rates of AEA. In the basal ganglia, higher expression of CB1 receptor has indeed been reported. Such alterations are linked with movement inhibition and might even be altered by persistent levodopa therapy. While some of these modifications that represent endogenous compensatory processes have been pointed to minimize the impact of DA failure in the basal ganglia, others would be likely to lead to the progression of typical motor symptoms of PD [97].

## 3. Alterations Observed in ECS and Basal Ganglia in PD

**Figure 2:**
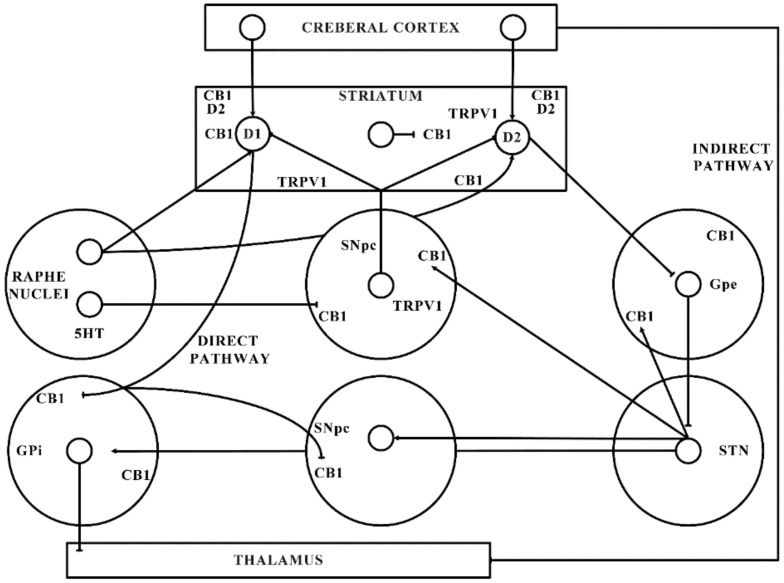
Basal ganglia organization. CB1—Cannabinoid 1 receptor; D1—Dopamine 1 receptor; D2—Dopamine 2 receptor; GABA—γ-aminobutyric acid; GPe—Globus pallidus external; GPi—Globus pallidus internal; SNpc—Substantia nigra pars compacta; STN—subthalamic nucleus; TRPV1—Transient receptor potential type-1.

The significant function of the ECS in PD is shown by the latest shreds of evidence gathered from various studies. The ECS elements are distributed strongly in the basal ganglia neural circuit (as presented in Figure 1) that is part of a dynamic neuronal network that connects bidirectionally with the glutamatergic, GABAergic, and dopaminergic signalling processes in the basal ganglia (Figure 2) [101].

Cannabinoids (CBs) play a prominent part in regulating communication of the striatal and cortical neuronal synapses, controlling the activation of a specific type of synaptic plasticity and altering motor functions [95]. The dopaminergic neuronal cell depletion occurs in PD, mainly results in reduced DA rates in striatum that leads to the alterations in the equilibrium between both the directly and indirectly acting eCB expression and basal ganglia pathways [102]. The foregoing cannabinoid signalling mechanism exhibits a biphasic transition pattern during PD progression [103]. Initially, presymptomatic and early stages of PD are marked by neuronal deterioration along with very little proof of cell death of neurons, which are correlated with CB1 receptor desensitization and exacerbation of cytotoxic damage (i.e., oxidative stress, excitotoxicity, and glial activation) [104]. The advanced/later and intermediate phases of PD are marked by extreme nigral deterioration and severe symptoms of PD, along with upregulated CB1 receptor activities and eCB ligands [104].

This might elaborate the capability of ligands with CB receptors to alleviate common PD symptoms. GABAergic neurons engages the internally and externally located parts of the SN and GP within the projecting GABAergic neurons of the brain, also regarded as medium spiny neurons (MSNs) that project to the nuclei of the basal ganglia and provide the striatal output via CB1 receptor expression [105,106]. The CB1 receptor is found in immune-reactive parvalbumin, interneurons, nitric oxide synthase (NOS) neurons, and cholinergic interneurons in the striatum [107]. On presynaptic axons, eCBs stimulate the CB1 receptor to diminish the release of neurotransmitters and glutamates and to serve as synaptic retrogressive messengers produced from post-synaptic neurons. In the same way, the CB1 receptor activation restrains both the release of GABA from striatal afferents and glutamate from cortex and thalamus [42,48]. CB1 presynaptic receptor activation in the outer parts of the GP may elevate the amount of GABA by minimizing its reuptake to the nucleus from striatal afferents and by lowering its release from striatal afferents in the SN as shown in Figure 2. Depending on all of these pieces of evidence, the activity of the neuronal basal ganglia system is considered to be regulated by the eCBs. The involvement of eCB signalling processes and their association with glutamatergic, GABAergic, and dopaminergic neurotransmitter signalling routes in various neuronal structures renders the ECS as a suitable prospective for a novel PD therapy [42,48,66].

## 4. Consequences of Basal Ganglia and Cortico-Striatal Plasticity in PD Associated Long Term Depression (LTD)

It is well established that synapses serve durable morphological and functional modulations throughout the basal ganglia neural circuit, especially in the striatum following the continuous stimulation of neuronal mechanisms. This interesting ability regarded as “synaptic plasticity” is assumed to take place at the levels of neuronal circuit dynamics and development, cortico-striatal synapses, and various key cognitive processes, mainly motor associated learning features [97,107]. DA plays a crucial role in creating two opposing kinds of synaptic cortico-striatal plasticity: long-term depression (LTD) and long-term potentiation (LTP). LTD renders glutamatergic synapses quite less excitable for potential future activation and LTP strengthens the connection between the cortical and striatal neurons. The LTP reversal is called depotentiation (LTP-D) and manages to adjust synaptic signalling to its natural state [97,108,109]. Although, LTD and LTP-D decrease the strength of synaptic signalling, depotentiation is itself unable to drag down non-potentiated synapses and necessitates the activation of *N*-methyl-d-aspartate (NMDA) receptors. Prior observations have already revealed that heterosynaptic LTP-D necessitates these receptors (CB1, adenosine A1, GABA-A, p38, mitogen-activated protein kinase (MAPK), and extracellular signal-regulated kinase (ERK) 1/2 signalling), implying that eCBs play a convoluted function in both pre- and post-synaptic changes [97,110,111,112,113]. In the mesencephalic brain areas, CB1 receptor activation has been shown to elevate the release of acetylcholine, thereby reducing the cholinergic deficit locally in PD [114]. Furthermore, CBs interact with the serotonergic system to influence the LID; loss of nerve supply in striatal dopaminergic renders a levodopa conversion that contribute to a non-physiologic throbbing DA release (false transmitter) [115]. As per one study, 2-AG metabolism takes place through the monoacylglycerol lipase (MAGL), the hydrolysing enzyme in this process. As per another experimental study, serine hydrolase ABHD6 (alpha/beta-hydrolase domain-6) knockdown has shown to decline the hydrolysis of 2-AG, influencing the migration of cells-induced by 2-AG stimulation in vitro [115,116]. In some studies, alteration in the synaptic plasticity (by genetic ablation of MAGL in mouse cerebellum and hippocampus) is reported to contribute to their desensitization through the mobilization of 2-AG and constant stimulation of CB1 receptor [40,117,118]. Similarly, the elevation of CB1-dependent LTD occurs by ABHD6 inhibition in mouse cortical excitatory synapses. ABHD6 also regulates the concentration of 2-AG that extends to the presynaptic CB1 receptor [116].

## 5. Experimental Studies Implying Potential Interventions of Cannabinoids as “Multi-Targeted” Compounds in Neuroprotection and Treatment of Motor and Non-Motor Complications

Clinically, CBs have been considered as neuroprotective agents because of their ability to act as anti-inflammatory agents, repress the calcium efflux and excitotoxicity, and exert antioxidative actions [119]. By mode of these two significant mechanisms, the CBs can impart neuroprotection in experimental PD models.

- Firstly, they diminish the augmented oxidative stress (OS) in PD, the process that appears to be irrespective of CB receptor participation.
- Secondly, by increasing the CB2 receptor’s density, particularly in activated microglia that controls the homeostasis of surrounding neurons and microfunctions of glial cells [14,38].

**Figure 3:**
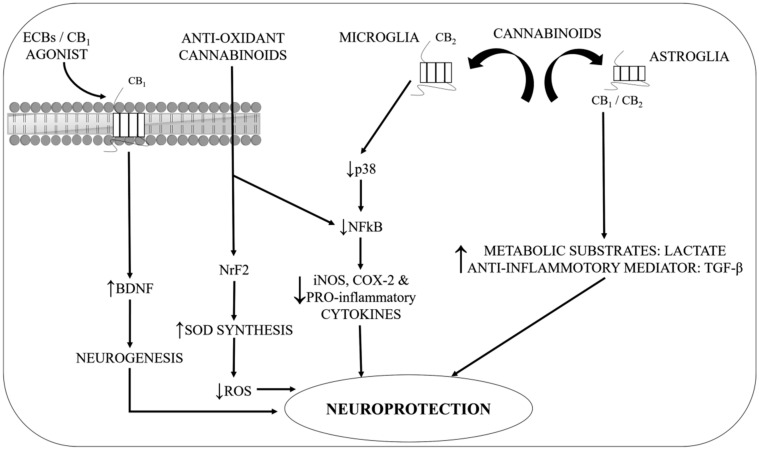
Probable mechanisms to demonstrate the neuroprotective action of cannabinoids in PD independent of CB-1R. BDNF—brain-derived neurotrophic factor; CB1—Cannabinoid 1 receptor; CB2—Cannabinoid 2 receptor; COX-2—Cyclooxygenase-2; iNOS—induced nitric oxide synthase; NFκB—nuclear factor-kappa B protein; NrF2—Nuclear factor erythroid 2-related factor; p38—mitogen-activated protein kinase; PRO-inflammatory—Proinflammatory; ROS—Reactive oxygen species; SOD—Superoxide dismutase; TGF-β—Transforming growth factor-β.

The neuroprotection is being provided by the phytocannabinoids; tetrahydrocannabinol (Δ9-THC) and CBD act against in vitro and in vivo toxicity of 6-hydroxydopamine (6-OHDA), such an activity being presumed due to enhancement of glial cell functioning [120,121]. As per numerous pieces of evidence, CBD was also able to recover and enhance 6-OHDA-induced DA reduction along with provoked Cu, Zn-superoxide dismutase upregulation, which is considered as being the principal enzyme against OS in the endogenous resistance [66,122]. CBs have not only been established to impart neuroprotection in cell tissue, but rather in rodent PD models too. One experimental study offered evidence in hemi parkinsonian rats of Δ9-THC that it acts as a neuroprotective agent. A notable recovery is observed in the dopaminergic transmission impairment produced by toxin upon prolonged administration of CB preceding the development of unilateral lesions of the dopaminergic neurons with 6-OHDA in the striatum, suggesting a reduction of dopaminergic cell death [40]. The data also propose that CBD declines the rise in the nicotinamide adenine dinucleotide phosphate (NADPH) oxidase expression and reduces the inflammation and oxidative damage indicators [123]. An alternative study had also highlighted the superoxide anion function caused by microglial NADPH oxidase in supplementing the dopaminergic neuronal demise (PD brain) [124]. Indeed, the process through which CBD performs its role in NADPH oxidase expression reduction is yet not verified but it is assumed that it occurs through CB1 or CB2 receptor interaction [125]. Although, information attained from various studies is the sign of the direct correlation between the mitochondrial functions and CB1 receptor in the brain [125], the decline in hydroperoxide-induced OS was produced by CBD and found to be protective against glutamate neurotoxicity, signifying CBD to be a strong antioxidant. Considering them all, these above findings assist the PD brain hypothesis that an antioxidant effect exerted by CB therapy can modify the generation of reactive oxygen species (ROS) in the mitochondria [66,126]. Besides, the neuroprotective effects regulated by the CB1 receptor modulation, the eCBs through their influence on the cells of the immune system such as B cells, T cells, neutrophils, monocytes, and granulocytes can also endeavour some prospective effects. During demonstration on both experimental and patient models of PD, it has been observed that neuroinflammation is a ubiquitous process. Besides the enormous loss of dopaminergic neuronal cells, it also manifests a prominent reaction of glial cells along with the neuroinflammatory responses shown by upgraded levels of cytokine and inflammatory-associated factors, considering here the inducible nitric oxide synthase (iNOS) and cyclooxygenase-2 (COX-2) as it is depicted in Figure 3 [127].

Thus, it is persuadable that CBs may affect the survival of neurons and conserve various regulatory synaptic functions during PD acting through CB1 receptor at “immune” CB2 receptor and “neuronal” receptors, delineating the strong pharmacological action to effect simultaneously, both synaptic and immune functions within CNS borders [127]. Altogether, the past and latest data encouraged the concept of the significant role of the endocannabinoid signalling in the motor control system [128,129]. Three significant pieces of evidence assist these findings.

- Firstly, there is indeed a strong involvement of TRPV1, CB1, and CB2 receptors linked with the cerebellum and basal ganglia eCBs that form the basis of regulating centres of movement.
- Secondly, there is proof of a potent inhibitory activity on the motor function of these endogenous, plant derivatives, and synthetic cannabinoids by optimizing the performance of numerous conventional neurotransmitters.
- Thirdly, there are significant alterations in the eCB transmission observed in human and PD animal models within basal ganglia.

These shreds of research evidence invigorate the notion that CBs perform the key processes in regulating ECS along with the transporters, receptors, and FAAH that could be therapeutically important due to their ability for curbing motor symptoms [130]. Besides, THC (the principal non psychotomimetic compound obtained from *Cannabis* species—CBD) has a weak affinity towards the CB receptor. Binding of the CBD at very low concentrations (in mM) with the CB receptor, serves its role as weak-potency agonist, antagonist, reverse agonist, or as a CB allosteric modulator of CB1 receptor. Some of these effects produced by CBD are counteracted by CB1 receptor reverse agonists, implying that this may produce “indirect agonism” on the CB1 receptor [23,131]. A multitude of in vitro studies have revealed idealistic neuroprotective results of CBD in PD models. In either of those models, CBD enhanced cell differentiation, expression, and viability of synaptic (synaptophysin and synapsin I) and axonal (GAP-43) proteins utilizing the PC12 cells and 1-methyl-4-phenylpyridinium (MPP+) treated SH-SY5Y cells. Such neuroprotective effects have relied on tropomyosin receptor kinase A (TrkA) receptor activation. Reduction of in cell viability is observed against lipopolysaccharide (LPS) and β-amyloid induced SH-SY5Y cells, depicting CBD as a neuroprotective agent. Alternately, in each of these rat primaries and N13 microglial cells, CBD-blunted ATP rises in the intracellular Ca^2+^ and also LPS-evoked production of nitrite. The authors proposed that the decrease of CBD encouraged the microglial cell stimulation based on CB and adenosine A1 receptors [132,133,134,135].

As suggested by observational studies, CBs may improve various PD-related motor and non-motor symptoms as discussed in Table 1. Multiple case series and single case reports reached the conclusion that CBs could possibly have favourable effect on motor symptoms of PD such as tremor, akinesia, or dyskinesia. In two published surveys, smoked cannabis has been confirmed to be beneficial for motor and non-motor symptoms in PD patients, along with several drawbacks that might have affected the results. No benefit to tremor was reported after administration of smoked cannabis as shown by small case series [97,136]. In comparison, improvement in bradykinesia, rigidity, sleep, tremor, and pain was reported in small open-label study, evaluating the motor exam after 30 min from administering smoked cannabis [97,137]. With respect to non-motor symptoms, a 4-week small open-label study of CBD for PD associated psychosis reported enhancement on the Brief Psychiatric Scoring Scale and Questionnaire on Parkinson’s Psychosis and also another case series observed potential benefits for rapid eye movement (REM) sleep behaviour disorder [97,138]. A small, 4-week randomized double-blind crossover study investigated the effect of Cannador, which failed to show an improvement in LID. In the most recent study, 21 patients with PD were randomized to placebo for a 6-week trial (CBD 75 mg per day or CBD 300 mg per day) [97,139]. Certain improvement was reported in the CBD (300 mg/day) group for the enhancement of the quality of life. Despite the low quality and sample size, the findings indicate that certain PD motor symptoms, particularly LIDs, may react to therapies based on cannabis. There were no serious adverse events reported and the side effects included dizziness, hypotension, visual hallucinations, vertigo, and somnolence [140].

### 5.1. Use of Cannabinoids in Reduction of LID: Implications of CB1 Agonists and Antagonists

Chronic levodopa administration, the principal treatment prescribed for motor symptoms in PD, can produce abnormally disabling involuntary movements, also called LID, in about 90% of patients. As, there are limited therapeutic approaches for the management of LID in PD, the CBs are important modulators and adjuncts to levodopa that helps to alleviate LID [144,145,146]. Various preclinical and clinical experimental evidences, as well as high expression of CB1 receptor regulating motor function in the brain areas, point towards eCB signalling as a pharmacotherapeutic target to treat motor disturbances associated with LID [146]. CB receptor stimulation improves GABAergic transmission in the GP by limiting the reuptake of GABA. A potential way to reduce LID could therefore be to increase GABAergic transmission in the GP by the CB receptor stimulation. This pilot study reveals that the nabilone, the CB receptor agonist can mitigate reuptake of GABA in the GP and ameliorate LID in PD [147]. In 6-OHDA-lesioned rat model, WIN55212-2 (a CB agonist) is subchronically administered at doses that do not affect DA-induced motor function and reduced LID unusual involuntary movements—a clinical LID behavioural correlation through a CB1-dependent pathway [146,148]. In the same model, WIN55212-2 also decreased hyperactivity of the LID protein kinase A, which is significantly linked with severity of dyskinesia [149]. In general, several studies indicate parkinsonism to be correlated with ECS signalling overactivity in the striatum, potentially delineating an endogenous compensatory mechanism that reflects a possible effort to stabilize striatal function subsequent to DA depletion. Afterwards, it was shown that ECS modulation could enhance parkinsonian symptoms in rodents as well as in primate PD experimental models [127]. In fact, in the 1-methyl-4phenyl-1,2,3,6-tetrahydropyridine (MPTP) marmoset PD model, there is an increase in the number of striatal CB1 receptors along with enhanced CB1 receptor G-protein coupling [150]. Nevertheless, higher CB1 receptor agonist doses may hinder motor function. In one study, the favourable effects failed to be reproduced by URB597, a FAAH inhibitor when administered alone. So, the FAAH inhibitor only exhibited antidyskinetic features in combination with an antagonist of the TRPV1 receptor, capsazepine [146,149]. Likewise, TRPV1 receptor stimulation by capsaicin or administration of URB597 alone has been shown to reduce hyperactivity caused by levodopa in reserpine-treated rats [151]. Furthermore, it is worth noting that CB1 receptors are expressed on serotonergic raphe fibres of striatum that can (1) lead to conversion of L-DOPA to DA and its release as a false neurotransmitter, thus promoting the LID development; (2) greatly affect the release of nigrostriatal DA; and (3) influence the control of glutamate release mediated by the DA and CB1 receptor [56,152,153,154]. We can thus theorize that CB agents can exhibit their antidyskinetic effects by diminishing the abnormal DA release from serotonergic terminals or/and by indirectly attempting to control the DA transmission [155].

Using various experimental PD models, preclinical studies have explored the role of both CB receptor agonists and antagonists, whether used alone or/and as co-adjuvants. The CB1 receptor agonists impedes the release of basal ganglia DA and are thus intended to be inadequate in ameliorating motor symptoms of PD. The bradykinesia was however exacerbated by CB1 receptor agonists in MPTP lesioned primates [156]. Therefore, various CB1 receptor agonists have been considered to enhance motor impairment, probably via non-dopaminergic processes, such as interactions with 5-hydroxytryptamine (5-HT) receptors and adenosine A2 receptors [157,158,159].

More consistently, CB1 receptor antagonist studies showed improvement in motor symptoms [160,161]. Blocking CB1 receptor with rimonabant, a CB1 receptor antagonist has shown a decrease in motor impairment and akinesia in experimental PD models, while some other studies presented contrary results [156,160,161,162]. In addition, rimonabant was even more effective at low doses and characterized by severe nigral damage in very advanced phases of this disease [161,162]. Such effects are assumed to involve the non-dopaminergic processes, including improved release of striatal glutamate [160,161]. These preclinical studies showed that antidyskinetic effects are manifested by both CB1 receptor agonists and antagonists.

### 5.2. Therapeutic Implications of Cannabinoids in Oxidative Stress during PD

An intensified rise in oxidative stress has previously been associated with PD. Mitochondria derived ROS are engaged in PD pathology as augmented oxidative markers wherein destabilized mitochondrial function renders neuronal damage in PD patients. The function of superoxide anion produced by NADPH oxidase in cellular and animal experimental studies emphasizes on rising dopaminergic neuron demise in PD. Recently detected lower CB1 receptors levels on the membrane of the mitochondria suggests a clear interaction between mitochondrial functions and the CB1 receptor in the brain wherein the phenolic moiety of CBs have been observed to impart antioxidant activity and protect against neurotoxicity induced by glutamate in a cellular model [12,124,125,163,164,165]. In rodents, recent evidence highlights that treatment with CBs can guard against the neuronal deterioration in cognitive impairment and diabetic neuropathy caused by experimental sepsis.

One recent study established the protective impact against the paraquat-induced ROS production by synthetic CBs [98,112,166,167]. Collectively, the hypothesis that CB therapy exerts antioxidant effects and may decrease the ROS production is backed by this research support in the PD brain mitochondria. Another research performed on C57BL/6J mice with neuropathy induced by cisplatin documented that the CBD declines the rise in expression of NADPH oxidase and declines inflammatory markers, and OS. Such a finding is particularly crucial as the hypokinetic activity of CBs, which regulates the CB1 receptor, implies a limitation for PD as these compounds shortly increase rather than declining the motor impairments. Correspondingly, prominent attempts are made for scrutinizing CB molecules that impart neuroprotection via antioxidant action, specifically activating the CB2 receptor and may counteract the CB1 receptor to favour the mitigating symptoms such as bradykinesia [165]. The direct influence of CBS on OS indicators has not been analysed but CB therapy turns away the decrease in cytosolic mRNA of endogenous antioxidant Cu-Zn superoxide dismutase leading to the intoxication of 6-OHDA.

Additional processes associated with the direct enhancement of native antioxidant enzymes include the stimulation of nuclear factor erythroid 2-related factor 2 (Nrf-2), the antioxidant transcription factor as shown in Figure 3 [165,168]. As per the latest study in BV-2 microglial cells, CBD was observed as an upregulating factor in the transcription of Nrf-2. Subsequently, the CBD also elevated the consequent downstream enzymes along with glutamate-cysteine ligase, heme oxygenase-1, glutathione S-transferase peroxidase, glutathione S-transferase, and NAD(P)H: quinine oxidoreductase, which serve its principal role in guarding against the electrophile and cytotoxic-induced OS. Nonetheless, in animal models, most of the robust data is required to assist the antioxidant ability of CBs. However, existing prior and recent findings did propose that treatment with CBs might serve as a remarkable tool for antioxidant therapy for treating PD and slowing down its progression [122,169].

### 5.3. Antidepressant and Analgesic Effect of Cannabinoids in PD

In Parkinson’s disease (PD), depression and pain are the common non-motor symptoms and despite their relevance, with the quality of life and poor health outcomes, patients are being underestimated, undertreated, and underdiagnosed. Pain, the relevant symptom is estimated to vary extensively with a mean prevalence of about or more than 50% and may result in psychological, social, and physical disorders and worsen the parkinsonian disabilities [66,167]. Distinctive therapies are utilized in the treatment of PD pain. Yet, the medications do not impart universal efficacy and demonstrate severe side effects. Cannabis is a plant widely known for its pain-relieving property and in modulating the perception of the pain via interaction between the CB receptors in the peripheral nervous system and CNS [66,170].

A multitude of preclinical and clinical studies have been carried out on marijuana, analysing its influence on pain. Smoked cannabis was found to significantly enhance physical disability, mood disturbance, and quality of life in HIV patients and to mitigate the neuropathic pain intensity. Cannabis was also efficacious in alleviating central and peripheral neuropathic pain in patients [171,172]. Relative to placebo, inhaled cannabis notably decreased the intensity of the pain (34%) in a clinical study of distal symmetric polyneuropathy (DSPN). *Cannabis sativa* (whole-plant extract) resulted in statistically notable improvements in the average pain intensity score. The medicinal therapy based on cannabis remarkably ameliorated the chronic pain severity along with the sleep disturbance in patients with multiple sclerosis. Oro-mucosal nabiximols in 1:1 combination of the CBD and THC reduced the pain intensity scores in neuropathic pain patients [66]. Such pieces of evidence are compatible with other findings, supporting the cannabis efficacy in decreasing the pain [66,173,174] and suggesting that CBs may also be effective in numerous disease phases including PD.

Various studies support the literature depicting the role of the endocannabinoid system in the mood and emotional behaviour control and blockade or the loss of embryonic stem cells (ESC) leading to depressive symptoms [175]. It has been shown that CB1 receptor antagonist, rimonabant induces these symptoms (depression or anxiety). Furthermore, gene polymorphism encoding the CB-2R has been linked with depression in PD patients. Low rates of THC produce elevated serotogenic activity and antidepressant activity through the CB1 receptor activation in animal models [66,176]. Additionally, different animal studies have shown that the antidepressive effect is exerted upon inhibiting the hydrolysis of the AEA that further leads to the elevation of the noradrenergic and serotonergic neuronal processes in the midbrain. Recent antidepressants act via raising the serotonin and/or noradrenaline levels, implying that ECS is a remarkable target for the novel antidepressants. Epidemiological shreds of evidence have revealed that the use of cannabis daily or weekly in people may exhibit a more positive effect and slighter depressed mood than the ones who do not use them. Numerous other studies have supported the link between depressive symptoms and heavy cannabis use but it is not clear if up surged depressive behaviour is based on the use of cannabis or other factors that raised the danger of heavy cannabis use and symptoms such as depression. Therefore, in PD patient, modest cannabis use might aid in ameliorating depressive symptoms and serve better life quality [66].

### 5.4. Therapeutic Implications of Cannabinoids in Excitotoxicity in PD

The literature has been widely supporting the association of excitotoxicity with PD. Parkin mediates the function and stability of “excitatory glutamatergic synapses” and this is widely supported by various studies. Postsynaptic expression of parkin impedes excitatory synaptic transmission that is expressed at postsynaptic areas and leads to pronounced loss of excitatory synapses within the neurons of the hippocampus. Contrary, an inadequate native parkin or parkin mutant expression is correlated to PD, which enhances the synaptic efficiency and stimulates glutamatergic synapses related elevated susceptibility to synaptic excitotoxicity. This exuberance of glutamatergic signalling may be a basis of excitotoxicity in the brain specifically SN. Repeated NMDA receptor stimulation raises intracellular calcium levels, producing an independent adjustment in concentrations of potassium, calcium, and sodium that harms ionic balance and results in serious cell swelling and death in PD [122]. Recently, the role of WIN-55,212-2 is explored by researchers in the dopaminergic neuronal demise stimulated by proteasomal synthase inhibitor (PSI) and its regulatory role in the accumulation of parkin and α-synuclein in the cytoplasm. It was observed that PC12 cells were guarded by WIN-55,212-2 against cytotoxicity induced by PSI via stimulating caspase-3. Contrarily, WIN-55,212-2 was shown to reduce the cytoplasmic aggregation of α-synuclein and parkin [122]. In contrast to prior reports, the CB1 receptor agonist was established to impede the activity of NF-κB. PSI in combination with WIN-55,212-2 was found to enhance the NF-κB nuclear translocation in the PC12 cells. Afterwards, it was observed that WIN-55,212-2 needs the protein presence, histidine triad nucleotide-binding protein 1 (HINT1) to counteract the harmful effects on the production of NO-mediated NMDA receptor and release of zinc. The above evidence indicates HINT1 as a peculiar protein needed for guarding the NMDA receptor-induced brain damage mediated by CBs [122,177]. Besides the classical CB agonist and antagonists, FAAH reversible inhibitor, AM5206, a novel class was explored and found to be a neuroprotective class against pre and postsynaptic proteins, against the kainic acid-induced excitotoxic harm to cultured hippocampal rats and slices in vivo and in vitro studies. This study encouraged the idea wherein protection is provided against acute excitotoxicity via eCBs. In an associated report, WIN-55,212-2 treatment was shown to impede the excitotoxicity in cultures of hippocampus and elevation of α-amino-3-hydroxy-5-methyl-4-isoxazole propionic acid (AMPA) receptor-induced by TNF-α [122,178,179,180]. The CB1 receptor prominence has been demonstrated in excitotoxicity circumstances (for example, rat striatum lesioned with the quinolinate and excitotoxin). Ameliorating the glutamate release is an eminent effect of CB agonists that would proclaim them as capable antiexcitotoxic substances for utilizing them in PD therapeutics. Such an effect was verified both in vitro and in vitro utilizing spinal cord neuronal cultures or other brain areas, and rodent models to induce ischemia respectively. Expression of the CB1 receptor takes place on two opposing synapses of neuronal populations (excitatory/glutamatergic and inhibitory/GABAergic) hence the activation of any or both of these might prompt dissimilar effects. Despite various shreds of evidence documented in the literature, the absolute mechanism involved in CB1 receptors that regulates the neuroprotection, is not yet known. In recent times, it was established that only the constricted CB1 receptor population found on glutamatergic endings are crucial to guard neurons against excitotoxicity [122,181].

### 5.5. Therapeutic Implications of Cannabinoids in Sleep Disturbances Associated with PD

Sleep disturbances are common among patients with PD and have an unfavourable effect on the quality of life with reported prevalence ranges from 25% to 98% [182]. There are multiple factors that potentiates PD sleep disorders including the drugs used to treat motor function and neurodegeneration [183]. Different sleep abnormalities have been described in PD patients including, insomnia, REM sleep behaviour disorder, sleep fragmentation, restless leg syndrome, excessive daytime sleepiness, and obstructive sleep apnoea [184,185]. CBDs along with two crucial phytocannabinoids, Δ9-THC and CBD, are being widely used as pharmacological agents for sleep disturbances among PD patients as they are considered to interact with ECS and other neurochemical pathways to influence circadian sleep/wake cycle, mood, autonomic function, and anxiety [186]. CBD, the crucial non-psychotic component of cannabis, has been demonstrated to improve REM sleep behaviour disorder in PD patients [138]. It has also been shown that marijuana improves the non-motor PD symptoms including sleep [137]. Nabiximols have been known to enhance subjective sleep parameters in clinical trials involving 2000 patients with different pain conditions [187]. Four PD patients in a small uncontrolled study with REM sleep behaviour disorder, received CBD (75 or 300 mg 99.9% purified) orally/day (dissolved in corn oil in the form of gelatine capsules) and observed diminished or completely eliminated nightmares, agitation, and kicking [114]. Thus, cannabis could be used to enhance PD patient’s life quality by ameliorating sleep disturbances and pain. 

Different PD models demonstrating the pharmacological impact of the cannabinoids are summarized in Table 2.

## 6. Δ9-THCV as a Promising Cannabinoid in the PD Treatment

Cannabinoid 2 receptor (CB2 receptor) agonist and antioxidant properties of the phytocannabinoid, Δ9-tetrahydrocannabivarin (Δ9-THCV) provided neuroprotection in PD experimental models whereas at low doses (≤5 mg/kg), its CB1 receptor antagonist afforded antihypokinetic effects. At the GPR55, it also seems to have some of the agonistic activity [195]. Using various experimental PD models, it was shown that attenuation of motor inhibition occurs in 6-OHDA-lesioned rodents by Δ9-THCV via low-dose CB1 receptor blockade, probably through alterations in glutamatergic transmission, alike the classic CB1 receptor antagonist, rimonabant. In LPS- and 6-OHDA-lesioned mice, it also preserved nigral neurons against certain degenerative stimuli to its CB-2R agonist and antioxidant properties [122]. The effects reported in 6-OHDA-lesioned mice were equivalent to those seen in CBD, showing antioxidant properties, unlike those found in LPS-lesioned mice, which were similar to those observed with a selective CB2 receptor agonist, HU-308 [196,197,198]. In the study, it was explored for the first time if a low dose Δ9-THCV is also antidyskinetic, a relevant antiparkinsonian property [195]. Earlier studies involved modulation of the ECS, specifically targeting the CB1 receptor in deferring LID [95,199]. To investigate the antidyskinetic potential of Δ9-THCV, a Pitx3ak mutant mice model, a relevant parkinsonism animal model linked with dopaminergic deficiency, was utilized, given the polymorphisms of Pitx^3ak^ has in fact been related to PD [200,201,202]. In the nigrostriatal system, Pitx^3ak^ mutant mice introduce a targeted bilateral depletion of dopamine associated with debilitated spontaneous locomotor action exhibiting akinesia. The presence of dyskinetic signs (horizontal and vertical hyperactivity) in the above model has been significantly prevented in the study by co-administration of Δ9-THCV, given the beneficial effect of antidyskinetic property. It is essential to note that the beneficial effect of Δ9-THCV had been reported when it was co-administered with levodopa over the entire treatment period, thereby reinforcing the concept that Δ9-THCV delays the development of LID [195].

## 7. Conclusions and Future Approach

This article reviewed crucial aspects of ECS in governing dopamine expression, synaptic plasticity, inflammation, and motor movement that enables CBs as an appropriate resource for researching neurodegenerative disease pathophysiology and investigating their ability for PD neuroprotection and pharmacotherapy. With PD becoming the second most persistent age-related neurodegenerative disorder, it is of utmost significance to discover effective treatment options for this pathology. The observation that substantial regulation of the cannabinoid signalling mechanism exists in PD has been supported by a large number of investigations. The above conceptions were strengthened by numerous preclinical and clinical findings. Thus, the pharmacological modification (with molecules that recognize specific and distinctive elements of cannabinoid signalling) could enhance motor behavioural anomalies as well as provide neuroprotection. Although cannabinoid-mediated mechanisms may not explicitly influence cell behaviour in tandem with their minimal interference in brain functions, optimizing the cannabinoid system could provide the greatest outcome in PD. The studies reviewed above reveal that cannabinoids may affect LID and PD progression. Several pathways tend to participate, varying from overt changes of vital neurotransmitters such as glutamate and dopamine to indirect anti-inflammatory activity. CBD appears as one of the most enticing medications in preclinical trials, among the cannabinoids probed so far. Selective counteraction of CB1 receptor and perhaps TRPV1 compounds may enhance motor disabilities—bradykinesia and LID. The antioxidant processes of some cannabinoid compounds, independent of their cannabinoid receptor activity, also have potential as a therapeutic treatment against PD development. A second aspect worthy of further exploration is the explanation of the activity and clinical applications of CB2 receptor in PD, as CB2 receptor agonists have mitigated the inflammatory reaction of microglia in PD. It is a “multi-target” compound, having a wide range of biological consequences in various neuropsychiatric disorders. Nonetheless, the particular function of this compound in the management of these conditions also needs to be established by broad and comparable clinical trials. It has been shown that the cannabinoid agonists and antagonists modify the ECS and alter motor behaviour. Antagonists of cannabinoid receptors tend to cause antiparkinsonian results whereas agonists of cannabinoid receptors possess a potent motor suppression that may be helpful in the treatment of motor complications. We also evaluated the role of the cannabinoid system and the constituents of marijuana in neuroprotection and found certain beneficial effects of marijuana. In the scenario, the optimal pharmacological compound should be sufficiently specific to attune the relationship between eCBs, DA, and glutamate in the striatum, permitting physiological synaptic plasticity and transmission without altering the interaction of the same system in other structures of neuronal basal ganglia.

## Figures and Tables

**Table 1:** Summary of various clinical study designs depicting whether cannabinoids enhance the motor or/and non-motor symptoms.

Study Design	Cannabinoids	Patients	Observations	References
Case series	Smoked cannabis, 1 g cannabis (2–9% THC)	5	No relief in tremors after one administration	[97,136]
Patient survey	Smoked cannabis	84	46% patients elaborate few benefits, 45% improvement of bradykinesia, 14% LID, 31% of rest tremor	[97,141]
Four-week open label	CBD up to 400 mg/day	6	Brief psychiatric rating scale improvement along with PD psychosis questionnaire	[97,142]
Patient survey	Cannabis	9	7 patients (78%) reported mood and sleep improvements, 2 patients improved motor symptoms	[97,143]
Randomized, double-blind, placebo-controlled	CBD 75 or 300 mg/day	21	Improvement for total PD psychosis questionnaire—39 score and daily activity sub-scores	[97,138]
Case series	CBD 75 or 300 mg/day	4	Improvements in rapid eye movement sleep behaviour disorder	[97,114]
Open label	Smoked cannabis, 0.5 g cannabis	22	Patients reported benefits for tremor, rigidity, pain, sleep, and bradykinesia (30 min after smoking cannabis)	[97,137]
Four weeks randomized, double-blind, placebo-controlled crossover	Cannador (1.25 mg CBD and 2.5 mg THC)	17	No benefits for LIDs on multiple outcomes	[97,139]

Legend: CBD—Cannabidiol; LID—Levodopa-induced dyskinesia; PD—Parkinson’s disease; THC—Tetrahydrocannabinol.

**Table 2:** A summarized form of different PD models demonstrating the pharmacological impact of numerous cannabinoids.

Compound	Model Involved	Activity Profile	References
OCE	A double-blind crossover study in dyskinetic patients	In PD patients, OCE was ineffective for treating levodopa-induced dyskinesia	[188]
WIN-55,212-2	PD model of L-DOPA—induced motor fluctuation	WIN-55,212-2 reduced AIMs to L-DOPA significantly by enhancing DARPP-32 and ERK1/2 phosphorylation in striatal neurons	[189]
OEA	6-OHDA PD mice model	OEA reduces symptoms and molecular markers of dyskinesia including the striatal overexpression of FosB.	[190]
HU-210 and WIN-55,212-2	LPS injection in rats intra-nigral	HU-210 and WIN-55,212-2 elevated the survival of the nigral neurons, inhibited the ROS generation, NADPH oxidase as well as pro-inflammatory mediators	[191]
THC	Lactacystin, MPP^+^, paraquat-induced neurotoxicity in SH-SY5Y cells	The neuroprotective effect is exhibited by THC by PPAR-γ receptor activation against all toxins	[192]
WIN-55,212-2	Proteasome inhibitors PSI-induced cytotoxicity in PC12 cells	PC12 cells are protected by WIN-55,212-2 and impede the cytoplasmic aggregation of α-synuclein and parkin	[193]
HU210 and AM251	L-DOPA-induced dyskinesia in the rat model	Subtypes of AIMs are significantly reduced by HU210 while no effect shown on AIMs by AM251	[194]
WIN-55,212-2	In 6-OHDA injected rats, AIMs induced by L-DOPA	WIN-55,212-2 mitigated L-DOPA induced AIMs	[149]
CBD, THC, THCA,	Cytotoxicity in mice mesencephalic cultures induced by MPP^+^	All exhibited antioxidative effects. Dopaminergic neurons protected by THCA and THC	[136]

Legend: 6-OHDA—6-Hydroxydopamine; AIMs—abnormal involuntary movements; AM251—1-(2,4-Dichlorophenyl)-5-(4-iodophenyl)-4-methyl-N-(1-piperidyl)pyrazole-3-carboxamide; HU-210—1,1-Dimethylheptyl-11-hydroxy-tetrahydrocannabinol; CBD—cannabidol; DARPP-32—Dopamine-and cAMP-regulated phosphoprotein; ERK—Extracellular signal-regulated kinase; FosB—G0/G1 switch regulatory protein 3 (G0S3); L-DOPA—Levodopa; LPS—Lipopolysaccharides; MPP^+^—1-methyl-4-phenylpyridinium; NADPH—Nicotinamide adenine dinucleotide phosphate; OCE—Oral cannabinoid extract; OEA—Oleo-ethanolamine; PC12—Cell line derived from a pheochromocytoma of the rat adrenal medula; PD—Parkinson’s disease; PPAR-γ—Peroxisome proliferator-activated receptor gamma; PSI—Proteasome inhibitor; ROS—Reactive oxygen species; SH-SY5Y—Human derived cell line; THC—Tetrahydrocannabinol; THCA—Tetra hydro-cannabinolic acid; WIN-55,212-2—(11R)-2-Methyl-11-[(morpholin-4-yl)methyl]-3-(naphthalene-1-carbonyl)-9-oxa-1-azatricyclo [6.3.1.04,12]dodeca-2,4(12),5,7-tetraene.
